# GENDER DIFFERENCES IN WIDOWHOOD IN THE SHORT RUN AND LONG RUN: FINANCIAL AND EMOTIONAL WELL-BEING

**DOI:** 10.1093/geroni/igz038.2698

**Published:** 2019-11-08

**Authors:** Jialu L Streeter

**Affiliations:** Stanford University, Stanford, California, United States

## Abstract

This paper addresses the question of whether differences in financial and emotional wellbeing after the death of one’s spouse vary systematically with gender, using the Health and Retirement Study data between 1992 and 2014. Results show that all else being equal, widows have 22% less income than widowers, and a nine percentage points increase in their likelihood of falling into poverty. The income gap attributable to gender is considerably large for those newly widowed. The gap shrinks by half in the next ten years in widowhood but widens again substantially after the ten-year mark. The study suggests that men are protected from income losses to a higher degree than women when they lose their spouse, and the survivor benefits of the Social Security and private pension plans do not necessarily cushion widows from this risk. We also find that, in widowhood, men experience more negative affects (e.g., depression and loneliness), while women face more challenges with somatic symptoms (e.g., restless sleep). The emotional gender gaps vary with widowhood durations. In the year of their spousal losses, men suffer significantly more than women from negative affects, in particular, loneliness. Though women score better than men in affects in the short run, they are prone to having more somatic symptoms in the long run, especially restless sleep. The results indicate that men and women need different types of emotional support and attention at different times of widowhood.

